# Calcific versus non-calcific plaque: a CAD-RADS and FFRCT study

**DOI:** 10.1007/s10554-024-03281-x

**Published:** 2024-11-21

**Authors:** David Murphy, John Graby, Benjamin Hudson, Robert Lowe, Kevin Carson, Sri Raveen Kandan, Daniel McKenzie, Ali Khavandi, Jonathan Carl Luis Rodrigues

**Affiliations:** 1 Cardiology Department, Royal United Hospitals Bath NHS Trust, Combe Park, Bath, Avon BA1 3NG UK; 2 Department of Health, University of Bath, Bath, UK; 3 Radiology Department, Royal United Hospitals Bath NHS Trust, Combe Park, Bath, Avon BA1 3NG UK

**Keywords:** Coputed tomorgaphy coronary angiography, Calcific coronary artery disease, Chronic coronary syndrome

## Abstract

Coronary Artery Disease-Reporting and Data System (CAD-RADS) standardises Computed Tomography Coronary Angiography (CTCA) reporting. Coronary calcification can overestimate stenosis. We hypothesized where CADRADS category is assigned due to predominantly calcified maximal stenosis (Ca+), the CTCA-derived Fractional Flow Reserve (FFRCT) would be lower compared to predominantly non-calcified maximal stenoses (Ca-) of the same CAD-RADS category. Consecutive patients undergoing routine clinical CTCA (September 2018 to May 2020) with ≥1 stenosis ≥25% with FFRCT correlation were included. CTCA’s were subdivided into Ca+ and Ca-. FFRCT was measured in the left anterior descending (LAD), left circumflex (LCx) and right coronary artery (RCA). Potentially flow-limiting classified as FFRCT≤0.8. A subset had Invasive Coronary Angiography (ICA). 561 patients screened, 320 included (60% men, 69±10 years). Ca+ in 51%, 69% and 50% of CAD-RADS 2, 3 and 4 respectively. There was no difference in the prevalence of FFRCT≤0.8 between Ca+ and Ca- stenoses for each CAD-RADS categories. No difference was demonstrated in the median maximal stenoses FFRCT or end-vessel FFRCT within CAD-RADS 2 and 4. CAD-RADS 3 Ca+ had a lower FFRCT (maximal stenosis p= .02, end-vessel p= .005) vs Ca-. No difference in the prevalence of obstructive disease at ICA between predominantly Ca+ and Ca- for any CAD-RADS category. There was no difference in median FFRCT values or rate of obstructive disease at ICA between Ca+ and Castenosis in both CAD-RADS 2 and 4. Ca+ CAD-RADS 3 was suggestive of an underestimation based on FFRCT but not corroborated at ICA.

## Introduction

Coronary Artery Disease (CAD) is characterised by atherosclerotic plaque accumulation within the walls of the major epicardial vessels [1]. The disease process is dynamic producing a spectrum of different disease entities including a longer lasting chronic coronary syndrome (CCS) interspersed with unstable episodes precipitating an acute coronary syndrome (ACS) [2]. CAD is an inflammatory process with mineralisation and subsequent calcification of the vessel wall representing the end stage [3]. Importantly both statin based therapy and exercise, have been shown to promote coronary calcification [4, 5], with such a CAD phenotype offering a degree of protection from spontaneous coronary events [6, 7].

A number of international guidelines advocate for the use of Computed Tomography Coronary Angiography (CTCA) as a diagnostic tool for patients presenting with clinical features of CCS [2, 8]. The Coronary Artery Disease Reporting and Data System (CAD-RADS) was first published in 2016 as a means of standardizing the reporting of CTCAs [9]. The CAD-RADS system subdivides arteries based on the maximal degree of stenosis and grades them from CAD-RADS 0 (no CAD) to CAD-RADS 5 (total occlusion).

However, CTCA is not without its challenges and one common factor impacting on the accuracy of CTCA is the presence of calcified plaque often resulting in blooming artefacts [10, 11]. This has been attributed to a number of factors but most importantly beam hardening and partial volume averaging. As the poly energetic beam from the CT pass through calcific disease low energy photons are attenuated thus “hardening” the beam [12] often seen as streak artefact. This beam hardening artefact leads to better penetration of more distal tissues thus lower attenuation values are recorded along this trajectory which may simulate non-calcific plaque. This results in a potential erroneous luminal assessment and overestimation of coronary stenosis leading to the potential for a false positive result [13]. Most modern scanners use filters to ‘pre-harden’ together with post-processing algorithms. The use of greater tube voltage can additionally help [12]. When tissues with distinct differences in densities and therefore absorption are encompassed by the same voxel the resultant averaged Hounsfield unit (HU) can again make interpretation difficult. The denser calcific component can falsely dominate the averaging. This may have the effect of overestimating the volume of calcific plaque. Better spatial resolution and convolution algorithms have meant a ‘sharper’ image is now possible [14]. In addition there has been an evolving learning curve and experience with cardiac CT technology and interpretation has improved with time.

CTCA provides a high diagnostic performance [15] and when combined with a non-invasively measured Fractional Flow Reserve (FFR_CT_) can now offer an accurate anatomical and physiological assessment of the major epicardial coronary vessels [16]. FFR_CT_ uses computational fluid dynamics (CFD), in this case blood, to calculate non-invasive FFR values [13]. A lower value indicating higher likelihood of flow limiting disease. Given the applications of Poiseuille’s law, small changes in the anatomical assessment of vessel boundaries will have significant impacts on resistance calculations and thus FFR_CT_ [17]_._ Calculations of FFR_CT_ therefore requires precise anatomical evaluation in a patient specific manner. This is not available through standard radiological reporting platforms and thus may provide a different anatomical interpretation to that of a reporting radiologist. CTCA images sent for FFR_CT_ undergo further processing including segmentation with sub voxel resolution techniques which have been shown to improve spatial resolution, as well as the use of millions of 3D sub-segmented volumes within the coronary tree, required for the resolution of fluid dynamic equations [18, 19].

It remains unclear as to the effect calcific disease can have on overestimation of luminal stenosis with modern CT imaging and algorithms. We hypothesized that where a CAD-RADS category was assigned due to a predominantly calcified maximal stenosis there would be an overestimation of luminal stenosis versus that of a non-calcific plaque. A subsequent physiological assessment by FFR_CT_ would therefore be expected to be lower compared to predominantly non-calcified maximal stenoses of the same CAD-RADS grade. A subset of these patients with an FFR_CT_ ≤ 0.8 also underwent an invasive coronary angiogram (ICA) and we hypothesised there would be a higher proportion with non-obstructive disease at ICA from stenoses that were predominantly calcified.

## Methods

### Patient population

**Fig. 1:**
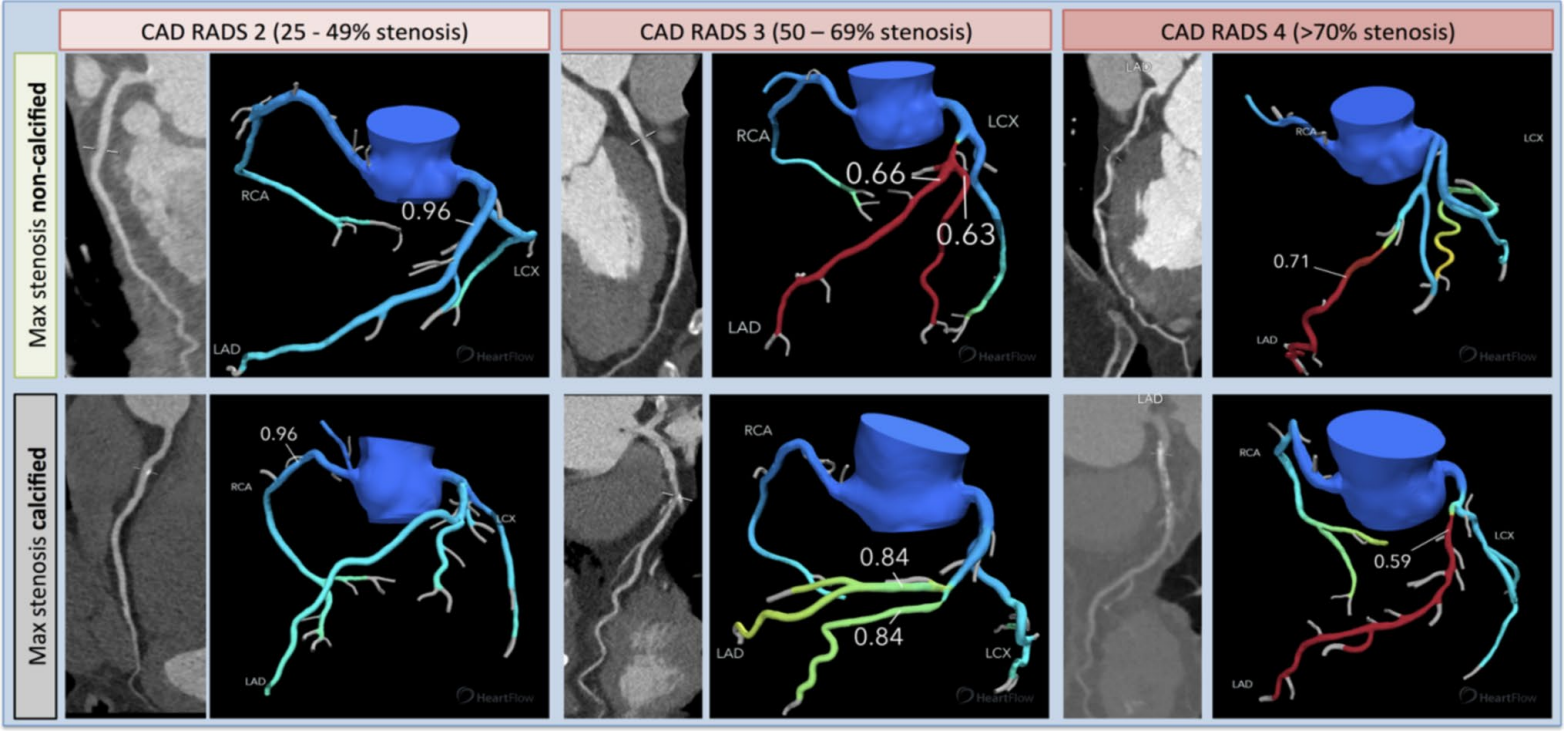
Representative examples of CAD-RADS categories subdivided by non-calcified maximal stenosis and calcified maximal stenosis with correlative FFR_CT_ values for the stenoses

This was a retrospective single-centre study of a prospectively maintained clinical database of concurrent patients who underwent investigation with a CTCA and FFR_CT_ from September 2018 to May 2020. Vessels not reported via the standardized CAD-RADS classification system were excluded, as were CAD-RADS 0 due to the absence of calcium and CAD-RADS 5 due to the lack of a comparator. CTCAs reported with a complete anatomical assessment of the right coronary artery (RCA), left circumflex (LCx) and left anterior descending artery (LAD) were divided by CAD-RADS categories 2 to 4 based on the maximal stenosis (CAD-RADS 2: 25%–49%, CAD-RADS 3: 50–69%, CAD-RADS 4: 70%–99%) for each patient and then subdivided by the presence or absence of calcified plaque (see Fig. 1). Calcified plaque was defined as high density tissue with density values greater than the contrast filled lumen occupying > 50% of the volume of the plaque by visual assessment [20]. Those patients where at least one vessel was reported as CAD-RADS N were then screened and those where an anatomical assessment was not possible due specifically to calcific disease were included in a separate category CAD-RADS N^C^.

FFR_CT_ data was provided by HeartFlow Inc. As previously published, CTCA images are used to construct anatomical modelling of the aortic root and coronary tree. A tetrahedral mesh then produces millions of data points allowing the computation of coronary pressure and flow using Navier–Stokes equations [21]. FFR_CT_ was measured 2 cm distal to the epicardial stenosis and end vessel FFR_CT_ was measured at the most distal point of the imaged vessel [22]. The likelihood of flow-limiting disease reported in line with published data (> 0.8 corresponding to a low likelihood, 0.75–0.8 borderline and < 0.75 to a high likelihood) [23]. Values reported as < 0.5 were given a value of 0.5 for statistical analysis. This patient cohort was also cross referenced with those that had undergone an ICA between September 2018 and May 2022 and findings compared on a per-vessel basis.

### CTCA acquisition

Studies were acquired with a 128-slice CT scanner (Siemens Edge, Siemens Healthineers, Erlangen). Imaging protocol consisted of a test bolus technique (12 ml Niopam 340 at 6 ml/sec) then full acquisition (60 ml Niopam 340 at 6 ml/sec) with a slice thickness of 0.6 mm, a pitch sequential scan feed of 34.5 mm, rotation time 0.28 s, and a tube voltage reference of 100kVp with automated kV modulation and 220 reference mAs with automated tube current modulation. Default CTCA reconstructions were: cardiac field of view 0.6 mm coronary vascular reconstruction kernel (I26f, Siemens Healthineers), 0.6 mm sharp vascular reconstruction kernel (I46f, Siemens Healthineers) and 0.6 mm raw axial reconstructions without automated smoothing between steps (Truestack I30f, Siemens Healthineers). Reporting was done by fellowship trained cardiothoracic radiologists with > 10 years experience.

### ICA

ICA performed were either on an Innova IGS 520 monoplane (GE Healthcare) or an Innova 2121 Biplane (GE Healthcare) with standard acquisition settings of 15 fps, 60 kV and 3.7 mA and fluoroscopy of 7.5 fps, 67 kV and 0.3 mA. Images were acquired according to standard practice with at least 2 projections obtained per vessel distribution. Angiographic functional assessment was incorporated in assessment at the discretion of the operator. This utilised a pressure wire (PW) study with an instantaneous wave free ratio (iFR) and/or fractional flow reserve (FFR) with end-vessel flow limiting disease cut-offs of ≤ 0.89 and ≤ 0.8 respectively [24]. A Volcano s5 imaging system with a Volcano Verrata Plus wire was used for both iFR and FFR measurements (Volcano Corp., Rancho Cordeova, California) intracoronary infusion of adenosine was also utilised for FFR measurements. ICA was undertaken by interventional cardiologists with > 10 years experience.

### Statistical analysis

Statistical analysis was performed using SPSS (Armonk, NY, USA: IBM Corp). A Shapiro Wilk test was used to assess for normality. Continuous variables are presented as a median ± standard deviation. Discrete variables are expressed as numbers and percentages. A Fisher’s exact test, Mann–Whitney *U* and Kruskal Wallis testing with post hoc Dunn’s test with Bonferroni correction were used where appropriate.

### Ethics

This study involved a retrospective review of clinically indicated diagnostics. As per the NHS Research Authority decision tool no written informed consent was obtained and no ethical committee approval was deemed necessary [25].

## Results

**Fig. 2:**
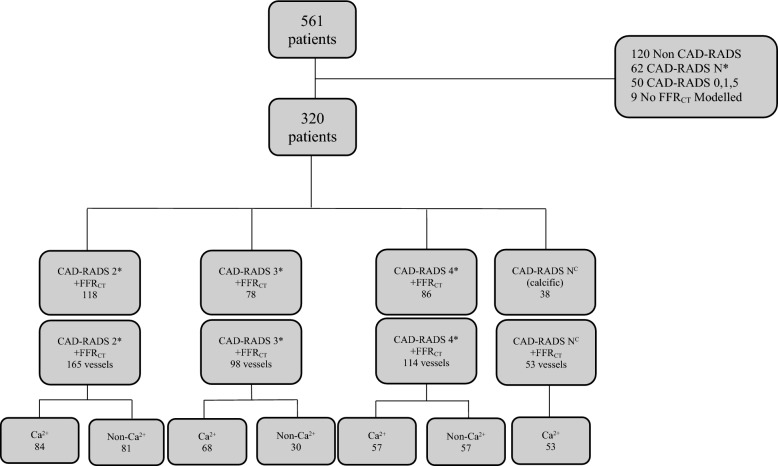
CAD-RADS categories subdivided by predominantly calcified or non-calcified maximal stenosis with their associated FFR_CT_. *Patients where any vessel had CAD-RADS N were excluded. *CAD-RADS N*^*C*^ CAD-RADS N where calcification resulted in the non-diagnostic classification, *CAD-RADS* coronary artery disease reporting and data system, *FFR*_*CT*_ non-invasive Fractional Flow reserve

561 patients were screened and 320 patients (430 vessels) were suitable for inclusion (60% men, median 70 years). A flow diagram is given in Fig. 2 and baseline study demographic details in Table 1.

**Table 1:** Study population demographics

Demographic	n = 320
Years of age (± IQR)	70 ± 14
Male gender (%)	190 (60)
Hypertension (%)	228 (72)
Diabetes Mellitus (%)	120 (38)
Dyslipidaemia (%)	92 (29)
Smoker (%)	109 (34)
Non-Smoker (%)	103 (32)
Ex-smoker (%)	106 (33)
Family history of premature CAD (%)	53 (17)
Typical angina (%)	132 (42)
Atypical angina (%)	121 (38)

*CAD* coronary artery disease

Predominantly calcified stenoses were present in 51%, 69% and 50% across CADS-RADS categories 2,3 and 4 respectively. The maximal stenosis was most commonly present in the LAD in all CAD-RADS categories. There was a difference in the proportion of calcific disease between CAD-RADS categories (2 vs 3 *p* = 0.004, 3 vs 4 *p* = 0.005, 2 vs 4 *p* = 0.9). Calcific artefact was specifically reported in 0%, 4% and 14% in CAD-RADS grade 2, 3 and 4 respectively.

There was a step-wise increase in the diagnosis of potentially flow-limiting disease, when assessed non-invasively through FFR_CT_, across the different CAD-RADS categories 2 to 4 (see Table 2). Median FFR_CT_ decreased with higher CAD-RADS categories (CAD-RADS 2 0.9 ± 0.05 vs CAD-RADS 4 0.7 ± 0.2, *p* < 0.01). Similar findings were seen with end-vessel FFR_CT_ (CAD-RADS 2 0.85 ± 0.06 vs CAD-RADS 4 0.66 ± 0.18, *p* < 0.01).

**Table 2:** FFR_CT_ results across CAD-RADS categories subdivided by calcified vs non-calcified maximal stenosis

	CAD-RADS 2 (n = 165)	Ca^2+^ (n = 84, 51%)	Non-Ca^2+^ (n = 81, 49%)	CAD-RADS 3 (n = 98)	Ca^2+^ (n = 68, 69%)	Non-Ca^2+^ (n = 30, 31%)	CAD-RADS 4 (n = 114)	Ca^2+^ (n = 57, 50%)	Non-Ca^2+^ (n = 57, 50%)	CAD-RADS N (n = 53)
FFR_CT_ ≤ 0.8 (n)	12	5	7	28	23	5	87	41	44	27
FFR_CT_ ≤ 0.8 (%)	7%	6%	9%	29%	34%	17%	76%	72%	77%	51%
Median FFR_CT_ (± IQR)	0.9 ± 0.06	0.9 ± 0.06	0.9 ± 0.05	0.86 ± 0.1	0.84 ± 0.13	0.89 ± 0.08	0.7 ± 0.15	0.74 ± 0.26	0.69 ± 0.3	0.8 ± 0.2
End vessel FFR_CT_ (± IQR)	0.85 ± 0.09	0.87 ± 0.08	0.85 ± 0.09	0.8 ± 0.12	0.79 ± 0.14	0.86 ± 0.1	0.66 ± 0.26	0.66 ± 0.26	0.64 ± 0.23	0.77 ± 0.18
Median LAD FFR_CT_ (± IQR)	0.9 ± 0.06	0.9 ± 0.06	0.9 ± 0.07	0.84 ± 0.13	0.82 ± 0.13	0.89 ± 0.08	0.7 ± 0.28	0.72 ± 0.25	0.7 ± 0.3	0.75 ± 0.22
Median LCx FFR_CT_ (± IQR)	0.88 ± 0.06	0.88 ± 0.08	0.89 ± 0.02	0.85 ± 0.1	0.84 ± 0.08	0.89 ± 0.3	0.74 ± 0.32	0.68 ± 0.27	0.68 ± 0.24	0.8 ± 0.25
Median RCA FFR_CT_ (± IQR)	0.92 ± 0.04	0.91 ± 0.05	0.92 ± 0.04	0.9 ± 0.04	0.9 ± 0.05	0.9 ± 0.06	0.68 ± 0.32	0.71 ± 0.36	0.68 ± 0.26	0.89 ± 0.1

*CAD-RADS* coronary artery disease-reporting and data system, *FFR*_*CT*_ non-invasive CT based fractional flow reserve, *SD* standard deviation, *LAD* left anterior descending artery, *LCx* left circumflex artery, *RCA* right coronary artery

38 patients were identified where ≥ 1 vessel had such significant calcification that luminal assessment was not possible (CAD-RADS N^C^) due to calcium induced artefact. This represented 12% of the patients included in the study. There were 3 patients within this group where all three major epicardial vessels were categorised as CAD-RADS N^C^. Overall 51% had potentially flow-limiting disease identified with a median FFR_CT_ 0.8 ± 0.14.

There was no difference in the prevalence of potentially flow-limiting disease (FFR_CT_ ≤ 0.8) between calcified and non-calcified stenoses for each CAD-RADS categories. Within CAD-RADS categories 2 and 4 there was no difference demonstrated in the median maximal stenoses FFR_CT_ or end vessel FFR_CT_ when subdivided into predominantly calcified and non-calcified maximal stenoses. There was a difference in CAD-RADS 3, Ca + 0.84 vs Ca- 0.89 (*p* = 0.02 for maximal stenosis and *p* = *0.0*05 for end-vessel FFR_CT_) with smaller numbers. Calcified stenosis resulting in a more ischaemic FFR_CT._

A total of 116 patients underwent an ICA, 4 where the maximal stenosis was graded as CAD-RADS 2 (median FFR_CT_ 0.88), 19 CAD-RADS 3 (median FFR_CT_ 0.75), 69 CAD-RADS 4 (median FFR_CT_ 0.68) and 24 where ≥ 1 vessel was graded as CAD-RADS N^C^. No statistical difference in the proportion of flow-limiting disease between calcified and non-calcified stenoses was found across categories 2,3 and 4 on a per vessel basis. CAD-RADS 2 was reclassified to unobstructed at ICA in 100% of cases, 73% in CADS-RADS 3 and 35% in CAD-RADS 4. Diagnosis of obstructive flow-limiting CAD was made by angiography alone in in 43% of cases in the CAD-RADS 2 category with 57% having angiography and subsequent invasive pressure wire assessment. This was 42% and 58% respectively in the CAD-RADS 3 category and 45% and 55% in CAD-RADS 4. ICA diagnosed flow limiting disease in 25% of the cases where ≥ 1 vessel was CAD-RADS N^C^ following CTCA, however this was on a per patient basis. On a per vessel basis the ischaemia categories based on FFR_CT_ were reclassified by ICA in 19%, see Table 3.

**Table 3:** Reclassification of vessels at ICA following an anatomic assessment of CAD-RADS N^C^ at CTCA

Modality	CTCA (CADS-RADS N^C^)		
ICA (LAD)	Category	FFR_CT_ ≤ 0.8	FFR_CT_ > 0.8
	Obstructive	7	1
	Non-obstructive	5	3
Modality	CTCA (CADS-RADS N^C^)		
ICA (LCx)	Category	FFR_CT_ ≤ 0.8	FFR_CT_ > 0.8
	Obstructive	9	0
	Non-obstructive	0	3
Modality	CTCA (CADS-RADS N^C^)		
ICA (RCA)	Category	FFR_CT_ ≤ 0.8	FFR_CT_ > 0.8
	Obstructive	1	0
	Non-obstructive	1	0

*CTCA* computed tomography coronary angiography, *CAD-RADS N*^*C*^ coronary artery disease-reporting and data system non-diagnostic (secondary to vessel calcification), *ICA* invasive coronary angiography, *LAD* left anterior descending artery, *LCx* left circumflex, *RCA* right coronary artery, *FFR*_*CT*_ non-invasive CT based fractional flow reserve

## Discussion

Our study did not demonstrate any statistical difference between FFR_CT_ values in both CAD-RADS 2 and 4 subdivided by calcific and non-calcific phenotypes. Only CAD-RADS 3 demonstrated a difference in both maximal FFR_CT_ stenosis and end vessel FFR_CT_. There was a lower FFR_CT_ value (more ischaemic) assigned to the calcific plaque implying there may be an underestimate of the degree of stenosis in this category rather than an overestimate as would be expected. In the sub-set of patients that underwent an ICA there was no statistical difference in the proportion of obstructive disease at ICA between calcified and non-calcified plaque types.

Calcification is often listed as a limitation of cardiac CT technology. Our study suggests some of the difficulties encountered with calcified lesions may now not be as significant as they were at the outset of CT imaging technology. This is an important consideration for physicians referring for diagnostic testing as the use of CTCA not only identifies all CAD, and therefore the ability to augment risk, but also leads to an improvement in the diagnostic angiogram to percutaneous coronary intervention (PCI) ratio relative to other diagnostics such as ischaemia testing. Likewise for those reporting cardiac CT an overestimation of the degree of stenosis based on the presence of calcification may lead to further downstream testing. This work would suggest that the anatomical read of CTCA is accurate and additional testing may not be required. Additionally even in cases where there was an inability to see the lumen due to calcific CAD a large proportion (75%) had no obstructive CAD at invasive angiography. Photon-counting CT has the potential to revolutionise the issue with both better spatial resolution and spectral imaging capabilities but currently the technology is very expensive and not widely available [26]. We routinely used a calcification specific reconstruction kernel which would be applicable in principle to all scanner types.

Vessels labelled as non-diagnostic (CAD-RADS N) at CTCA may be seen as a failure from a radiological reporting point of view but it must be remembered that calcific disease is pathognomonic of CAD so the non-diagnostic aspect simply relates to the degree of stenosis. Following the work of trials such as ORBITA [27] and ISCHAEMIA [28] guidelines have become increasingly orientated towards medical therapy in the first instance with ICA offered for refractory symptoms, thus potentially limiting the absolute necessity to have a grade of luminal stenosis for every CTCA. All of these patients had successful FFR_CT_ evaluation thus providing physiological data. Additionally identification of significant vessel calcification may be helpful for any downstream PCI is undertaken. The imaging may provide an opportunity to better plan the intervention such as the need for calcium modification techniques or additional procedure length.

In addition to being a single centre retrospective study this work had a number of limitations. Firstly the calcific plaque morphology such as density or circumferential arc were not assessed. We chose to compare calcified and non-calcified lesions defined by the calcific volume rather than density as clinically this is measured more frequently in routine care. Previous work, however, has demonstrated no impact on sensitivity, specificity or diagnostic accuracy across quartiles of calcium density as assessed by the Agatston score [29]. Any additional impact of geometric parameters other than the degree of stenosis, such as lesion length, was again not factored into the work.

For those patients that underwent ICA no formal quantitative coronary angiography (QCA) was undertaken and any functional assessment was at the discretion of the operator, as this study was based on routine clinical care. The rationale for referral for an ICA was also not examined in this paper. Ongoing symptoms despite medical therapy, patient preference or clinician preference would likely contribute to selection bias. The CAD-RADS N^C^ category may be particularly affected by this as the majority had only one vessel in this category on a per patient basis so the rationale for undertaking an ICA may be have been based on a high CAD-RADS category in another vessel.

Previous literature has predominantly focused on the ICA as a comparator, with many older studies utilising quantitative coronary angiography (QCA) with a cut off of ≥ 50% to indicate obstructive disease [30–32]. This negates the fundamental difference between the modalities in that ICA is a luminogram whereas CTCA is not. More recent studies have utilised intravascular imaging to overcome such discrepancies [13]. From a methodological perspective this is a more robust approach but a statistical difference between the modalities may not translate to a clinically important effect. Day to day CTCA is predominately used for its sensitivity, minor discrepancies in lumen diameter or calcification length may well only be important clinically if PCI is being planned based on the technology. Additionally such a comparison requires an invasive assessment. FFR_CT_ technology has been previously extensively validated (against invasive assessments) including the ADVANCE registry [33], NXT trail [16], DEFACTO [34] and DISCOVER FLOW [35]. Furthermore Uzu et al. [36] undertook work looking specifically at the ability to accurately measure FFR_CT_ based on OCT measured minimum lumen area (MLA) and comparing luminal boundaries. In vessels > 1 mm there was no impact on accuracy.

## Conclusion

Overestimation of vessel stenosis due to calcific CAD may be less relevant with modern sharp kernel reconstruction techniques. CAD-RADS N due to obscuration of the lumen by calcific disease remains problematic in modern practice.

## Data Availability

No datasets were generated or analysed during the current study.
